# Psychometric properties of the Brazilian version of the “Caregiver Guilt Questionnaire” (CGQ-BR)

**DOI:** 10.1590/1980-5764-DN-2025-0325

**Published:** 2025-11-14

**Authors:** Katia Carreira Pfutzenreuter, Andrés Losada-Baltar, María Márquez-González, Deusivania Vieira da Silva Falcão

**Affiliations:** 1 Universidade de São Paulo, Departamento de Gerontologia, São Paulo SP, Brazil.; 2 Universidad Rey Juan Carlos, Department of Psychology, Madrid, Spain.; 3 Universidad Autónoma de Madrid, Biological and Health Psychology Department, Madrid, Spain.

**Keywords:** Alzheimer Disease, Caregivers, Guilt, Validation Study, Doença de Alzheimer, Cuidadores, Culpa, Estudo de Validação

## Abstract

The “Caregiver Guilt Questionnaire” CGQ was originally developed in Spain and contains 22 self-report items covering the following factors: guilt about doing wrong by the care recipient, guilt about failing to meet the challenges of caregiving, guilt about self-care, guilt over feeling negative emotions while caregiving, and guilt about neglecting other relatives.

**Objective:**

To analyze the psychometric properties of the Brazilian version of the CGQ.

**Methods:**

The sample was established for convenience, consisting of 153 family caregivers of people with Alzheimer’s disease. The following measures were applied: a sociodemographic questionnaire, the CGQ, the Depression, Anxiety and Stress Scale (DASS-21), and the Life Satisfaction Scale. An exploratory factor analysis was performed for analyzing construct validity to obtain evidence of validity with internal structure and external correlation measures.

**Results:**

The exploratory factor analysis identified five factors with 22 items, which explained 71% of the total variance. Criterion validity was supported by positive associations between CGQ-BR scores and levels of depression, anxiety, and stress, and a negative association with life satisfaction. Good to excellent reliability indexes were found for the total score and subscales.

**Conclusion:**

The psychometric properties of the CGQ-BR, when applied to a sample of family caregivers of people with Alzheimer’s disease in the Brazilian population, proved to be a valid tool for measuring feelings of guilt. The findings suggest that this tool can be used in clinical and research settings with caregivers.

## RESUMO

O “Caregiver Guilt Questionnaire” (CGQ) foi originalmente desenvolvido na Espanha e contém 22 itens de autorrelato que abrangem os seguintes fatores: culpa por fazer algo errado pelo receptor do cuidado, por não conseguir enfrentar os desafios de cuidar, culpa sobre o autocuidado, por sentimentos negativos durante a prestação de cuidados e pela negligência de outros familiares.

**Objetivo:**

Analisar as propriedades psicométricas do CGQ para a população brasileira.

**Métodos:**

A amostra foi estabelecida por conveniência, constituída por 153 cuidadores familiares de pessoas com doença de Alzheimer. Foram aplicados: questionário sociodemográfico, Questionário de culpa do cuidador, Escala de depressão, ansiedade e estresse (DASS-21) e Escala de satisfação com a vida. Foi realizada uma análise fatorial exploratória para analisar a validade do construto para obter evidências de validade com estrutura interna e medidas de correlação externa.

**Resultados:**

A análise fatorial exploratória identificou cinco fatores com 22 itens, que explicaram 71% da variância total. A validade de critério foi evidenciada por associações positivas entre os escores do CGQ-BR e os níveis de depressão, ansiedade e estresse, e por uma associação negativa com a satisfação com a vida. Índices de confiabilidade bons a excelentes foram encontrados para o escore total e subescalas.

**Conclusão:**

As propriedades psicométricas do CGQ-BR, quando aplicado a uma amostra de cuidadores familiares de pessoas com doença de Alzheimer na população brasileira, mostraram-se uma ferramenta válida para mensurar sentimentos de culpa. Os achados sugerem que esta ferramenta pode ser utilizada em cenários clínicos e de pesquisa com cuidadores.

## INTRODUCTION

Alzheimer’s disease (AD) affects approximately 32 million individuals worldwide, with an additional 69 million in the prodromal phase and 315 million in the preclinical phase, totaling over 416 million people across the various stages of the disease^1^. In Brazil, underdiagnosis rates reach nearly 80%, and most individuals receive a diagnosis only at an advanced stage of the disease^2^. This situation reflects multiple factors, including insufficient public investment in professional training within the Unified Health System (SUS), limited public awareness, and persistent stigma surrounding aging and dementia, which often leave families assuming full caregiving responsibilities without adequate support or resources^3^.

Caring for a person with dementia often has a significant impact on a caregiver’s mental health. Although there are positive aspects, such as feeling useful or giving meaning to one’s life, what is most reported in the scientific literature are the negative impacts on the physical and mental health of this population, which include stress, depression, and anxiety^4,5^. Among the emotional effects that come with caregiving, guilt is an emotion that involves feelings of failure regarding a personally relevant moral or social standard; it is associated with an individual’s personal context and beliefs about caregiving^6^.

The emergence of guilt in caregivers of individuals with dementia may be influenced by three central aspects:

- the perceived moral obligation to assume responsibility for the care of the person with dementia;
- the tendency to view one’s caregiving performance negatively, interpreting actions as potentially harmful or inadequate toward the care recipient;
- feelings of having neglected personal needs or other life domains as a consequence of the demands of caregiving^7^.

The feeling of guilt has been the subject of international studies and presents a challenge to caregivers as it is not always a conscious feeling and often goes unrecognized. There is also a tendency for caregivers to deny their experience or confuse it with similar feelings^8^. In these cases, the questionnaires help health professionals to accurately determine and identify the existence and severity of a caregiver’s emotional state. Previous cross-sectional and longitudinal research has found support to the importance of guilt as a potential emotional state that significantly contributes to caregivers’ distress levels^9^, with the study of guilt being considered an emerging topic in the dementia field^10^.

More recently, researchers have demonstrated that caregiver guilt contributes to depressive symptoms both directly and indirectly through psychological flexibility processes, including cognitive fusion, experiential avoidance, and difficulties in values-based action^11^. Consistently, latent profile analyses have identified subgroups of caregivers characterized by distinct emotional vulnerability patterns, where the presence of rigid beliefs about care, high levels of guilt, ambivalence, and experiential avoidance are linked to greater psychological distress and desire for institutionalization^12^. These findings highlight the clinical relevance of guilt and psychological flexibility, emphasizing the need for accurate assessment and targeted interventions.

The “*Caregiver Guilt Questionnaire*” (CGQ) is a tool that assesses the feelings of guilt for dementia caregivers, although it is also used for measuring this emotional state in other caregiving populations, such as caregivers of people with cancer^13^ or heart failure^14^. It was originally developed and validated in Spain with a sample of family caregivers of people with dementia^6^.

The questionnaire contains 22 self-report items covering the following five factors of guilt:

- guilt about doing wrong by the care recipient;
- guilt about failing to meet the challenges of caregiving;
- guilt about self-care;
- guilt about neglecting other relatives;
- guilt about having negative feelings towards other people.

The CGQ was replicated and validated in the United Kingdom and Portugal with satisfactory reliability and validity indexes, including the same 5-factor structure as the original scale^15,16^.

Predicated on validation studies of this instrument in Spain, the United Kingdom and Portugal, our aim is to analyze the psychometric properties of the CGQ in a Brazilian population of primary family caregivers of individuals with Alzheimer’s disease and related dementias (ADRD). Considering the distress associated with guilt and the large number of individuals providing care for a family member with ADRD in Brazil, it is essential to have a valid instrument available to assess guilt in Brazilian Portuguese. Accordingly, the objective of this study is to present the psychometric properties of the CGQ for the Brazilian population.

## METHODS

### Participants and procedures

Participants were recruited using a non-probabilistic convenience sampling strategy. Study invitations were disseminated via social media platforms (e.g., Instagram, WhatsApp) and through collaborations with organizations that support family caregivers of individuals with Alzheimer’s disease. Data collection was conducted online between April and June 2023. Eligible participants were community-dwelling primary family caregivers of individuals diagnosed with ADRD. Inclusion criteria were:

- age≥18 years;
- Brazilian nationality;
- self-identification as the primary family caregiver of an individual with ADRD;
- cohabitation with the care recipient;
- provision of daily caregiving for at least six months prior to enrollment;
- provision of informed consent.

Exclusion criteria included:

- being a secondary family caregiver;
- institutionalization of the care recipient;
- refusal or withdrawal of consent at any stage of the study.

All study materials were approved by the Ethics Committee of the Universidade de São Paulo (USP) (Certificate of Presentation for Ethical Appreciation—CAAE: 62501922.1.0000.5390). The present adaptation and validation of the CGQ were carried out in collaboration with the original developers of the instrument.

### Measures

A socio-demographic questionnaire was filled out by caregivers to determine their age, gender, marital status, religion, level of education, degree of kinship, number of children (if any), how long they have been caregivers, whether they rely on professional assistance to help with caregiving duties, seek psychological counseling, self-assessed opportunities for leisure activities (“To what extent do you have leisure activity opportunities?” [1—Not at all; 2—Very little; 3—Medium; 4—Very much; 5—Completely]) and self-assessment of health status (“How satisfied are you with your health?” [1—Not at all; 2—Very little; 3—Medium; 4—Very Much; 5—Completely]).

The CGQ^6^ is a 22-item self-report developed in Spain that measures the caregiver’s day-to-day feelings and thoughts, with a 5-factor structure, also validated in the United Kingdom and Portugal^15,16^. It focuses on the individual’s previous few weeks and is measured on a four-point Likert frequency scale where never=0, rarely=1, sometimes=2, several times=3, and always or almost always=4. The Brazilian adaptation of CGQ was conducted directly from the original Spanish version following international guidelines for cross-cultural adaptation^17^. The full transcultural adaptation process, including forward and back-translation, expert panel review, and pilot testing with Brazilian caregivers, has been previously published^18^.

The Depression, Anxiety and Stress Scale (DASS-21) in this study is a 21-item self-report tool that measures three related negative emotional states (depression, anxiety and stress). It uses a Likert scale ranging from 1 (totally agree) to 5 (totally disagree). It was developed by Lovibond and Lovibond^19^ and adapted into Portuguese for adults, older adults and adolescents in order to investigate the validity and reliability of this instrument^20^. In this sample, the anxiety factor presented Cronbach’s Alpha (α=0.83) and McDonald’s Omega (ω=0.83), the depression factor presented Cronbach’s Alpha (α=0.89) and McDonald’s Omega (ω=0.90), and the stress factor presented Cronbach’s Alpha (α=0.90) and McDonald’s Omega (ω=0.90).

The “Satisfaction with Life Scale” measures one’s overall perceived satisfaction with life. It consists on a 5-item Likert format ranging from 1 (I totally agree) to 5 (I totally disagree). This scale was developed by Diener et al.^21^ and adapted for Brazil by Gouveia et al.^22^. In this sample, the scale demonstrated good internal consistency, with Cronbach’s Alpha (α) and McDonald’s Omega (ω) both equal to 0.86.

### Data analysis

#### Exploratory factor analysis

The data matrix was generated through polychoric correlations. Sampling adequacy and factorability were assessed using the Kaiser-Meyer-Olkin test (KMO; above 0.60) and relevance from Bartlett’s Test of Sphericity. Standardized factor loadings of ≥0.30 were considered relevant. These tests were used to determine the number of factors based on classical parallel analysis^23^. The minimum residual method of factor analysis was used and the oblique oblimin rotation was applied.

#### Internal consistency

Reliability was assessed through Cronbach’s Alpha and McDonald’s Omega indexes, with indexes ≥0.70 considered satisfactory in exploratory studies, scores between 0.80 and 0.90 considered good and higher than 0.90 excellent. Coefficients were calculated for the different dimensions.

#### Associations between variables

A correlation analysis was performed between the total score and factors of the CGQ, DASS-21, the Satisfaction with Life Scale, and two follow-up questions on opportunity for leisure activities (on a Likert scale ranging from 1=not at all to 5=completely) and satisfaction with health (on a Likert scale ranging from 1=very dissatisfied to 5=very satisfied). The analyses were tested using the JASP software (version 17.2). Spearman’s rank-order correlation was employed due to the ordinal nature of the variables and the presence of non-normal distributions. This method is also more appropriate for variables measured on ordinal scales, such as self-rated health and opportunities for leisure activities, for which Pearson’s correlation would not be suitable.

#### Group comparisons

To examine whether there were significant differences in the CGQ global score across religious groups (no religion, catholic, evangelical, spiritist, other) and relationship to the care recipient (mother, father, spouse, third-degree relative), an analysis of variance (ANOVA) with Welch’s correction was employed as a more robust alternative to the traditional F-test^24^. If a significant difference was found, bootstrapping procedures (1,000 resamples; 95% BCa confidence intervals) would be applied to enhance the reliability of pairwise comparisons and provide more robust confidence intervals for the differences between means^25^. Omega squared (ω^2^) was used to estimate the overall effect size^26^.

## RESULTS

### Sample characteristics

A total of 153 family caregivers of a relative with ADRD participated in the study (92,15% women; M=51 years, standard deviation—SD=9,53; range: 21–74), most of whom were married or in a stable relationship (52,93%). In relation to religious beliefs, 43.13% (n=66) identified as Catholics, 18.3% (n=28) as Atheists, 12.41% (n=19) as evangelicals, 16.34% (n=25) as Spiritists, and 9.8% (n=15) as other. Most caregivers were adult children caring for their mothers (71.89%; n=110), and had been providing care for more than five years (40.52%; n=62). The majority (60%; n=85) reported not relying on professional help to assist them in caring for people with Alzheimer’s disease, 51.63% (n=79) worked outside the home, and 65.35% (n=100) did not seek or receive psychological counseling. Table 1 presents the characteristics of the participants.

**Table 1:** Sociodemographic and caregiving characteristics of the sample (N=153).

		n	(%)	M (SD) / Range
Caregiver age (years)		153		51.0 (9.53) / (21–74)
Caregiver sex	Female	141	92.15	
	Male	12	7.84	
Marital Status	Married / Stable relationship	81	52.93	
	Single / Divorced	67	43.78	
	Widowed	5	3.26	
Religious Affiliation	Catholic	66	43.13	
	Atheist	28	18.3	
	Spiritist	25	16.34	
	Evangelical	19	12.41	
	Other	15	9.8	
Relationship to Care Recipient	Mother	110	71.89	
	Third-degree relative	17	11.11	
	Father	16	10.45	
	Spouse/Partner	10	6.53	
Duration of Caregiving	>5 years	62	40.52	
	3–4 years	31	20.26	
	2–3 years	23	15.03	
	1–2 years	22	14.37	
	<1 year	15	9.8	
Professional Caregiving Assistance	No	85	60	
	Yes	68	40	
Employment (working outside the home)	No	74	48.37	
	Yes	79	51.63	
Receiving Psychological Support	No	100	65.35	
	Yes	53	34.65	
Geographic Region (Brazil)	Southeast	91	59.47	
	South	39	25.49	
	Northeast	18	11.76	
	Central-West	4	2.61	
	North	1	0.65	

### Descriptive statistics of Caregiver Guilt Questionnaire items

The data set was initially analyzed to check for any inconsistencies and/or missing values related to the participants’ responses to the items. Table 2 presents descriptive and asymmetry and kurtosis data for the original 22 items of the CGQ.

**Table 2:** Descriptive analysis for the original 22 items in the Brazilian Version of the Caregiver Guilt Questionnaire.

Item	Average score	SD	Asymmetry	Kurtosis	Min Range	Max Range
1	1.90	1.12	0.15	-0.44	0.00	4.00
2	1.98	1.07	0.04	-0.54	0.00	4.00
3	1.93	1.18	-0.10	-0.72	0.00	4.00
4	1.92	1.12	-0.12	-0.61	0.00	4.00
5	2.06	1.17	-0.04	-0.65	0.00	4.00
6	1.13	1.02	0.53	-0.51	0.00	4.00
7	1.88	1.25	0.16	-0.96	0.00	4.00
8	1.97	1.11	0	-0.50	0.00	4.00
9	1.73	1.07	0	-0.55	0.00	4.00
10	2.22	1.09	-0.27	-0.44	0.00	4.00
11	1.95	1.17	-0.13	-0.81	0.00	4.00
12	1.92	1.34	0.01	-1.10	0.00	4.00
13	2.10	1.03	-0.03	-0.42	0.00	4.00
14	1.93	1.22	-0.04	-0.91	0.00	4.00
15	1.98	1.19	-0.17	-0.77	0.00	4.00
16	1.44	1.28	0.53	-0.72	0.00	4.00
17	0.55	0.92	1.83	3.04	0.00	4.00
18	1.37	1.44	0.55	-1.11	0.00	4.00
19	1.63	1.34	0.31	-1.05	0.00	4.00
20	1.41	1.25	0.41	-0.86	0.00	4.00
21	1.11	1.05	0.56	-0.63	0.00	4.00
22	1.23	1.10	0.70	-0.09	0.00	4.00

Abbreviations: SN, standard deviation; Min., Minimum; Max., Maximum.

Notes: The Caregiver Guilt Questionnaire (CGQ) is composed of five factors: Factor 1=Guilt about doing wrong by the care recipient; Factor 2=Guilt about not rising to the occasion as caregivers; Factor 3=Guilt about self-care; Factor 4=Guilt about neglecting other relatives; Factor 5=Guilt about having negative feelings towards other people.

Source: adapted from Losada et al.^6^.

### Internal structure

Bartlett’s Test of Sphericity was significant (χ^2^=2,916.34, df=231, p<0.001), and the KMO measure of sampling adequacy was 0.87, both indicating that the correlation matrix was suitable for factor analysis. A classical parallel analysis^27^ suggested the retention of five factors (Supplementary Material — available at https://www.demneuropsy.org/wp-content/uploads/2025/08/DN-2025.0325-Supplementary-Material.docx). For analysis purposes, the factorial model with five factors was used. The factor loadings for the CGQ are shown in Table 3.

**Table 3:** Factor loadings of the Caregiver Guilt Questionnaire items.

CGQ items	Factors				
	1	2	3	4	5
10. I have felt bad about getting angry with the person I’m caring for.	0.96				
14. I have felt bad about not having more patience with the person I’m caring for.	0.91				
11. I have felt bad about telling off the person I’m caring for, for some reason.	0.84				
12. I’ve got angry with myself for having negative feelings toward the person I’m caring for.	0.78				
8. I have felt bad about things I may have done wrong with the person I’m caring for.	0.58				
2. I have felt guilty about the way I’ve sometimes behaved with my relative.	0.54				
13. I’ve found myself thinking that I’m not up to the job.	0.36	0.32			
21. I have thought that the way I care for my relative may not be appropriate and may make his/her problem get worse.		0.86			
5. I have thought that I’m not doing things right with the person I’m caring for.		0.82			
9. I have thought that perhaps I’m not caring well for my relative.		0.70			
22. I have felt guilty thinking that my lack of information and preparedness might mean that I’m not handling the care of my relative in the best way possible.		0.66			
6. I have thought that, given the circumstances, I’m doing a good job as a caregiver.		0.57			
7. When I’ve gone out to do some pleasant activity (e.g. eating out in a restaurant), I’ve felt guilty and unable to stop thinking that I should be caring for my relative.			0.84		
16. I have felt bad for leaving my relative in the care of someone else while I had fun.			0.74		
1. I have felt bad about having made some plan or done some activity without taking my relative into account.			0.57		
15. I have felt bad about leaving my relative in the care of someone else while I do my own things (e.g. work, shopping, going to the doctor).			0.49		
18. I have felt like a bad person for hating and/or envying other relatives who could have taken responsibility for some caring and do not do so.					0.93
19. I have felt bad for having negative feelings (e.g. hate, anger or resentment) toward some relatives.					0.78
17. I have felt guilty about having wished that others “could have this burden” or suffer as I do.					0.59
20. I have felt guilty about having so many negative emotions in relation to caring.				0.42	
3. I have felt bad for not looking after my other relatives (husband, wife, children…) as I should, due to my caregiving.				0.96	
4. I have felt bad about not being able to devote more time to my family (husband, wife, children…), due to my caregiving.				0.94	

Notes: 1. The oblimin rotation method was applied. Total variance: 71%. 2. Items are grouped into five factors: Factor 1=Guilt about doing wrong by the care recipient; Factor 2=Guilt about failing to meet the challenges of caregiving (includes item 13, due to theoretical considerations); Factor 3=Guilt about self-care; Factor 4=Guilt about neglecting other relatives (includes item 20); Factor 5=Guilt about having negative feelings towards other people.

Source: adapted from Losada et al.^6^.

As shown in Table 3, all the items have factor loadings over the recomended score (0.30) and were grouped into five factors that correspond to the factors obtained in the original CGQ study^6^:

- guilt about doing wrong by the care recipient;
- guilt about failing to meet the challenges of caregiving;
- guilt about self-care;
- guilt about neglecting other relatives;
- guilt about having negative feelings towards other people.

The total explained variance was 71%.

Item 13, “I think I am having trouble coping with caregiving duties”, presented a cross factor loading in both factor 1 and factor 2. We decided to retain Item 13 given its theoretical importance of describing a behavior. We decided to leave it in factor 2 (guilt about failing to meet the challenges of caregiving) due to its similarity with the other items and the theoretical construction of the factor, in addition to maintaining the original structure of the CGQ.

### Internal consistency

Cronbach’s Alpha and McDonald’s Omega reliability coefficients were calculated as follows: Factor 1 (α=0.92; ω=0.93); Factor 2 (α=0.86; ω=0.87); Factor 3 (α=0.83; ω=0.84); Factor 4 (α=0.82; ω=0.85); and Factor 5 (α=0.91; ω=0.91); and the reliability index for the global scale (22 items) (α=0.93; ω=0.93).

### Correlation analysis

Spearman’s correlations between the CGQ global score and factors and all the assessed variables are shown in Table 4.

**Table 4:** Correlations between Caregiver Guilt Questionnaire global score and factors and the assessed variables.

Scales		r		p-value
Total Score	Stress	0.62	***	<0.001
Total Score	Anxiety	0.52	***	<0.001
Total Score	Depression	0.47	***	<0.001
Total Score	Satisfaction with life	-0.26	**	<0.01
Total Score	Questions about leisure activities	-0.26	**	<0.01
Total Score	Satisfaction with health	-0.31	***	<0.001
Factor 1 Score	Stress	0.61	***	<0.001
Factor 1 Score	Anxiety	0.45	***	<0.001
Factor 1 Score	Depression	0.46	***	<0.001
Factor 1 Score	Satisfaction with life	-0.24	**	<0.01
Factor 1 Score	Leisure activities	-0.23	**	<0.01
Factor 1 Score	Satisfaction with health	-0.32	***	< 0.001
Factor 2 Score	Stress	0.45	***	<0.001
Factor 2 Score	Anxiety	0.36	***	<0.001
Factor 2 Score	Depression	0.34	***	<0.001
Factor 2 Score	Satisfaction with life	-0.18	*	0.03
Factor 2 Score	Satisfaction with health	-0.19	*	0.02
Factor 3 Score	Stress	0.35	***	<0.001
Factor 3 Score	Anxiety	0.33	***	<0.001
Factor 3 Score	Depression	0.26	**	<0.01
Factor 3 Score	Leisure activities	-0.19	*	0.02
Factor 3 Score	Satisfaction with health	-0.19	*	0.02
Factor 4 Score	Stress	0.59	***	<0.001
Factor 4 Score	Anxiety	0.55	***	<0.001
Factor 4 Score	Depression	0.50	***	<0.001
Factor 4 Score	Satisfaction with life	-0.29	***	<0.001
Factor 4 Score	Leisure activities	-0.20	*	0.01
Factor 4 Score	Satisfaction with health	-0.26	***	<0.001
Factor 5 Score	Stress	0.34	***	<0.001
Factor 5 Score	Anxiety	0.32	***	<0.001
Factor 5 Score	Depression	0.30	***	<0.001
Factor 5 Score	Leisure activities	-0.31	***	<0.001
Factor 5 Score	Satisfaction with health	-0.29	***	<0.001

Notes: *p<0.05

**p<0.01

***p<0.001.

As shown in Table 4, significant associations between the CGQ total and factor scores and all the assessed variables were found, except for the associations between factor 2 (guilt about failing to meet the challenges of caregiving) and leisure activities, and factor 3 (guilt about self-care) and 5 (guilt about having negative feelings towards other people) with satisfaction with life.

### Group comparisons

No significant differences were found in the global score across religious groups (F[4, 148]=0.71, p=0.58; ω^2^=0.00). The effect size was small, suggesting limited practical relevance of the group differences. Significant differences were found in the global score based on the caregiver’s relationship to the care recipient (F[3, 149]=2.99, p=0.03; ω^2^=0.04). Post hoc test revealed a statistically significant difference between the mother and third-degree relative groups (ΔM=11.64, 95% BCa CI [3.98–17.76]; p=0.03; d=0.72), indicating a higher mean score in the mother group. The other comparisons did not show statistically significant differences: mother vs. father (ΔM=5.77, 95% BCa CI [-4.14–14.05]; p=0.56), mother vs. spouse (ΔM=5.99, 95% BCa CI [-8.01–13.24]; p=0.73), father vs. spouse (ΔM=0.23, 95% BCa CI [-14.38–10.94]; p=1.00), father vs. relative (ΔM=5.73, 95% BCa CI [-3.65–17.27]; p=0.71), and spouse vs. third-degree relative (ΔM=5.76, 95% BCa CI [-3.43–19.69]; p=0.77; d=0.38).

## DISCUSSION

The aim of this study was to obtain evidence of the psychometric properties of the Brazilian version of the Caregiver Guilt Questionnaire (CGQ-BR) scale based on internal structure, reliability, and association with other relevant variables. As this study aimed to adapt the instrument to a new cultural context, exploratory factor analysis (EFA) was employed in accordance with recommendations by Orçan^28^, who highlights the importance of using EFA prior to confirmatory factor analysis (CFA) in scale adaptation studies to detect potential structural changes arising from linguistic or cultural differences. The division of the 22 items into five5 factors was in line with previous psychometric studies of the CGQ done in Spain, UK and Portugal^6,15,16^. The total explained variance of 71% obtained in this study was satisfactory and superior when compared to the development study, which presented a total explained variance of 59.25, and 60% that was obtained in the UK and 65.8% in Portugal^15,16^.

Item 16 was removed from Teixeira’s study^16^ and most of the items had been grouped differently as compared with the Spanish^6^ and UK^15^ versions; however, in this study, the items were grouped similarly to the scale development and validation done in the United Kingdom. In this study, the only item with a cross-factor loading was Item 13 (“I think I am having trouble coping with caregiving duties“), which presented a cross-factor loading in both factor 1 and factor 2. We decided to retain Item 13 given its theoretical importance of describing a behavior and the fact that it presented a higher factorial load in factor 2.

Item 20 (“I have felt guilty about having so many negative emotions in relation to caring”) loaded in factor 4 (“guilt about neglecting other relatives”) as it was obtained for the United Kingdom^15^ (located in factor 3, “guilt about self-care”) and Portugal^16^ (located in factor 2, “guilt about failing to meet the challenges of caregiving”) versions. This is different from the original study where it was retained in factor 1 (“guilt about doing wrong by the care recipient”). The other items were retained in the same factors as the instrument development study.

Moderate to low associations were found between the assessed variables following Mukaka’s^29^ criteria, with the largest associations found between CGQ total score and depression, anxiety and stress. Regarding the association with other relevant variables for the caregiving field, the CGQ and its factors showed positive associations with depressive, anxiety, and stress symptoms, coherent with the findings that show that guilt among caregivers is associated with anxiety, depression and stress^6,12,13^. In addition to these findings in validation studies, other studies have also shown significant associations between these variables, indicating that higher levels of guilt are also associated with higher levels of anxiety, depression and stress^9,11,12,30^.

On the other hand, negative significant associations have been found between the CGQ total and factor scores and satisfaction with life, opportunity for leisure activities, and satisfaction with health, as theoretically predicted in the study of the development of the CGQ^6^ and validation studies^15,16^. The findings obtained with a Brazilian sample of family caregivers of people with dementia are coherent with previous studies that found that caregivers who have higher levels of guilt tend to have a more negative perception of satisfaction with health and life, as well as a lower frequency of leisure activities^31^.

In the group comparisons, no significant differences were found in guilt scores across religious affiliations, suggesting that, in this sample, religious background may not strongly influence caregiver guilt. In contrast, caregivers of mothers reported significantly higher guilt levels compared to those caring for more distant relatives. This finding aligns with previous studies highlighting the particular vulnerability of filial caregivers to guilt, due to stronger emotional bonds, cultural expectations, and moral obligations related to parental care^7,11,32,33^. These results emphasize the importance of considering relational contexts when evaluating caregiver guilt.

This study has several limitations, including the cross-sectional nature of the study. Future longitudinal or experimental studies are needed in order to provide additional support for the use of the Brazilian version of the CGQ. In addition, although the sample size can be considered small for doing psychometric analysis, the coherent results with previous studies of the development^6^ and validation studies to other languajes of the CGQ^15,16^ provide empirical support to the findings of this study. Further studies may benefit from wider and more heterogeneous samples, including caregivers or people with other dementias or other illnesses (e.g., caregivers of people with cancer or schizophrenia). Future research may further strengthen the current findings by employing larger and more diverse samples. Such studies could include CFA to validate the underlying structure of the constructs assessed in this study.

In spite of the mentioned limitations, the data obtained in this study suggest that the CGQ-BR shows precision and good to excelente reliability, with a high explained variance and good correlation data with external measures, findings that recommend its use for research and clinical purposes with Brazilian family caregivers of people with dementia.

## Data Availability

The datasets generated and/or analyzed during the current study are available from the corresponding author upon reasonable request.
